# Hyperalgesia when observing pain-related images is a genuine bias in perception and enhances autonomic responses

**DOI:** 10.1038/s41598-019-51743-3

**Published:** 2019-10-24

**Authors:** Anaïs Chapon, Caroline Perchet, Luis Garcia-Larrea, Maud Frot

**Affiliations:** 1 NeuroPain lab, Neurosciences Research Center of Lyon, INSERM U1028 Bron, France; 2 University Claude Bernard Lyon 1, Bron, France

**Keywords:** Sensory processing, Neurophysiology

## Abstract

Observing pain in others can enhance our own pain. Two aspects of this effect remain unknown or controversial: first, whether it depends on the ‘painfulness’ of the visual stimulus; second, whether it reflects a genuine bias in perception or rather a bias in the memory encoding of the percept. Pain ratings and vegetative skin responses were recorded while 21 healthy volunteers received electric nociceptive shocks under three experimental conditions: (i) observing a painful contact between the body and a harmful object; (ii) observing a non-painful body contact, (iii) observing a control scene where the body and the object are not in contact. Pain reports and vegetative responses were enhanced exclusively when the subjects observed a painful body contact. The effect on perception was immediate, abated 3 sec after the shock, and positively correlated with the magnitude of vegetative arousal. This suggests that (a) hyperalgesia during observation of painful scenes was induced by their pain-related nature, and not by the simple body contact, and (b) hyperalgesia emerged from a very rapid bias in the perceptual encoding of the stimulus, and was not the result of a retrospective bias in memory recollection. Observing pain-depicting scenes can modify the processing of concomitant somatic stimuli, increasing their arousal value and shifting perception toward more painful levels.

## Introduction

Pain perception is sensitive to the context where the pain occurs. While deflecting attention toward other sensory stimuli can decrease pain sensations, observing someone else suffering can enhance our own pain^1–3^, decrease pain tolerance and, in a subset of subjects, even induce a physical pain sensation^4–6^. These effects are considered independent from mere unpleasantness or disgust, since unpleasant or disgusting stimuli unrelated to human pain failed to modulate it^2^.

In healthy subjects, conscious pain perception is correlated with vegetative reactions, mainly sympathetically-mediated, such as the skin sympathetic response (SSR)^7,8^. Such vegetative reactions are objective signals that co-vary with both subjective pain perception and brain activity^9,10^, and represent reliable indexes of arousal that the subject cannot manipulate voluntarily^8,9^. Enhancement of these autonomic responses has been reported during observation of other’s pain^11–13^, and was found to be correlated with the ability to develop empathic behaviour^11^. Moreover it has been shown that such vegetative responses are modulated by the same high-order areas involved in empathy-induced hyperalgesia, in particular ventromedial prefrontal structures^14,15^. While it is reasonable to consider a link between the modulation of vegetative changes and that of subjective pain perception during observation of others’ pain, such specific relationship has not been, to the best of our knowledge, studied so far.

In this study we assessed the relation between vegetative and pain perceptual changes during presentation of a validated set of pictures that are able to induce pain-related subjective reactions. Our aim was to address several issues that have not been explored previously. First, the robustness of the hyperalgesia that can be driven by pain observation was tested by using a set of images depicting painful situations with lower emotional impact than those we and others have used in previous studies. Thus, the target pictures represented human body parts seen from a first-person perspective in occasional contact with harmful objects^16^ but without additional emotional attributes (no blood, no injuries, no facial expressions of pain…). Secondly, in order to assess the specific effects of the painful attributes of the images, rather than the mere observation of object-to-body contacts, the present study integrated control pictures identical to the ‘painful’ images, but with a non-aggressive object instead of the painful one (e.g. a feather replacing a hammer). Indeed, it has been reported that the simple observation of objects touching the body is able to induce both vicarious sensations and brain activity changes^17,18^. Finally, we tested whether hyperalgesia induced by the observation of other’s pain could be attributed to a genuine modification in its perceptual encoding^2^ rather than a bias in the short-term memory encoding of the stimulus. Indeed, in most previous experiments (including ours), the subjects’ pain ratings came only after a delay of several seconds relative to the actual delivery of the stimulus, making it impossible to disentangle perceptual from working memory biases. We therefore compared changes in pain ratings reported either immediately or several seconds following stimulus presentation.

## Results

### Behavioral data

**Figure 1:**
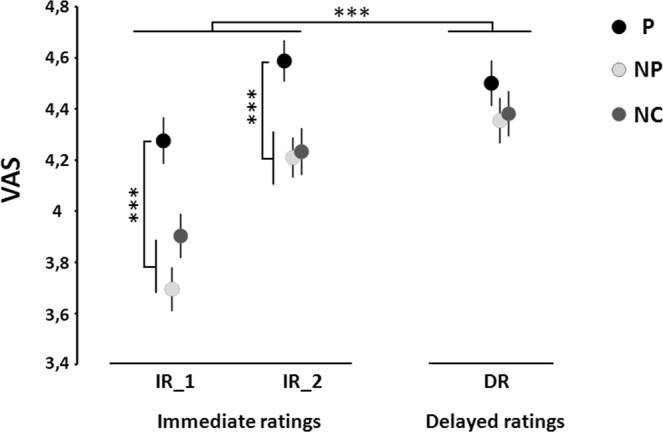
Mean ratings of electrical shocks delivered after the presentation of pictures illustrating a painful contact (P), a non-painful contact (NP) and no contact (NC) between an object and a body part in three rating conditions. IR: immediate ratings, DR: delayed ratings. ***p < 0.001.

A two-way ANOVA was performed on pain ratings with “image type” [Pain (P) *vs*. No-Pain (NP) *vs*. No-Contact (NC)] and “block type” [immediate 1 (IR_1) *vs* immediate 2 (IR_2) *vs* delayed (DR)] as factors (Fig. 1). ANOVA analysis showed a significant effect of image type on the pain ratings to electrical shocks (*F*_(2,20)_ = 15.1; *p* < 0.001), a significant effect of block type (F_(2,20)_ = 23.2; *p* < 0.001), and a significant image * block type interaction (F_(2;20)_ = 4.09; *p* = 0.003) (Fig. 1). Global effect size calculated from the ANOVA (Cohen’s d) was 0.57. Post-hoc analyses showed significantly higher intensity ratings for shocks that followed pain-depicting images only for the two blocks with the immediate ratings condition (For IR_1: P *vs* NP, t_(2,20)_ = 6.8, p < 0.001; P *vs* NC, t_(2,20)_ = 5, p < 0.001; For IR_2: P *vs* NP, t_(2,20)_ = 5.3, p < 0.001; P *vs* NC, t_(2,20)_ = 4.8, p < 0.001). No significant difference emerged when comparing the two types of non-painful pictures (For IR_1: NP *vs* NC, t_(2,20)_ = −1.7, ns; For IR_2: NP *vs* NC, t_(2,20)_ = −0.3, ns). Although a similar trend of average ratings was also observed in the delayed situation (3 sec after the shocks), the difference did not reach statistical significance (Fig. 1 and Table 1) (P *vs* NP, t_(2,20)_ = 1.8, ns; P *vs* NC, t_(2,20)_ = 0.9, ns; NP *vs* NC, t_(2,20)_ = −0.5, ns). Ratings were compared between the DR block *versus* the two others IR blocks by using an Helmert contrast analysis, which showed that pain ratings to all the conditions were higher in the DR than in the two IR blocks (p < 0.001).

**Table 1:** Pain ratings obtained for the three different images types in the three block sessions: Immediate Ratings 1 and 2 (IR_1, IR_2) and Delayed Ratings (DR).

Pain ratings		Block types		
		IR_1	IR_2	DR
Image types	Painful	4.28 ± 0.09	4.59 ± 0.08	4.5 ± 0.09
	Non-Painful	3.7 ± 0.09	4.21 ± 0.08	4.35 ± 0.09
	No Contact	3.9 ± 0.09	4.23 ± 0.09	4.38 ± 0.09

In order to estimate the magnitude of the hyperalgesic effect observed in the immediate rating conditions, the ratings in the two ‘non painful’ conditions (NP and NC pictures) were pooled together and used as reference ratings for each of the two IR blocks. The enhancement of pain ratings during the pain-picture condition was of 15.3 ± 5.3% for IR_1 and 10.5 ± 1.8% for IR_2. The difference between the two immediate conditions was not significant (t = 0.8, ns).

### SSR amplitudes and latencies

**Figure 2:**
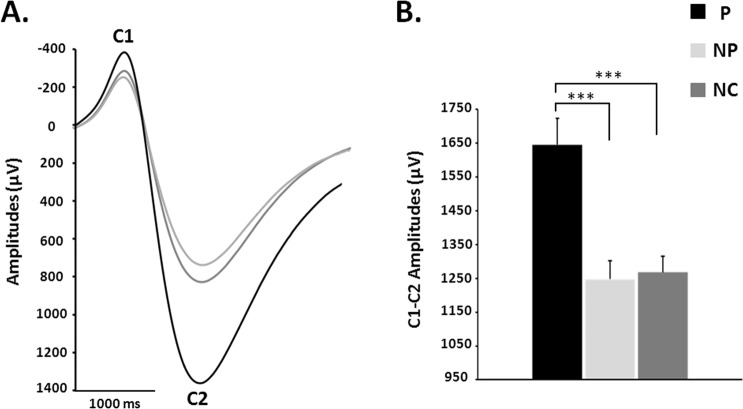
(**A**) SSR averages elicited by pictures illustrating a painful contact (P), a non-painful contact (NP) and no contact (NC) between an object and a body part. For illustration purposes, SSR elicited by the three image types were realigned so that the first component C1 was at the same latency; therefore the latency values are not represented on the figure. (**B**) Peak to peak C1-C2 amplitudes of the SSR elicited by the three different image types. ***p < 0.001.

SSR amplitudes were significantly affected by the picture type (F_(2,20)_ = 18.3; *p* < *0.001*). Global effect size calculated from the ANOVA (Cohen’s d) was 0.3.Bonferroni-corrected post-hoc analyses showed SSR amplitude to be significantly enhanced for P pictures (P: 1645.3 ± 78.3 µV), relative to both NP (NP: 1247.8 ± 53.9 µV; t _(2,20)_ = 4.7, *p* < 0.001) and NC pictures (NC: 1268.3 ± 48 µV; t _(2,20)_ = 4.8, *p* < 0.001), while no difference was shown between the two latter image types (t _(2,20)_ = −0.2, *ns*) (Fig. 2). If the SSR associated to all the ‘non painful’ images (NP and NC) were pooled together and considered as a “reference” SSR, the SSR amplitude enhancement was of 32.7% between this reference and the pain-picture condition.

**Figure 3:**
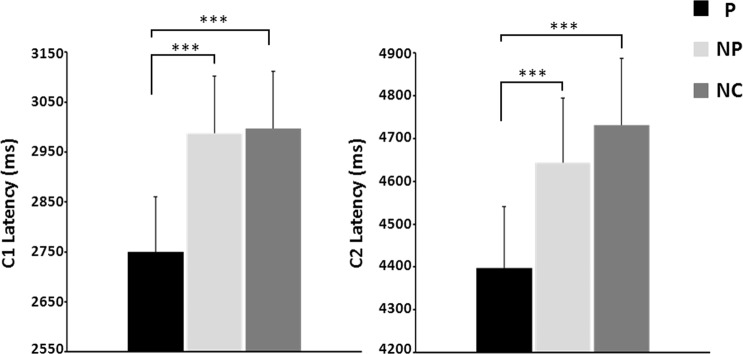
Latencies of the negative C1 and positive C2 components of the SSR elicited by pictures illustrating a painful contact (P), a non-painful contact (NP) and no contact (NC) between an object and a body part. ***p < 0.001.

ANOVA also showed a significant effect of the picture type on the latencies of the C1 and C2 components of the SSR (for C1: *F*_(2,20)_ = 15.3; *p* < 0.001; for C2: *F*_(2,20)_ = 14.6; *p* < 0.001; effect size d = 0.3) (Fig. 3).

For C1 component, Bonferroni-corrected post-hoc analyses showed significantly shorter latencies for P pictures (P: 2770.8 ± 32.8 ms), relative to both NP (2970.3 ± 33.7 ms; t_(2,20)_ = −4.4, *p* < 0.001) and NC pictures (2951.1 ± 34.5 ms; t_(2,20)_ = −5.2, *p* < 0.001). No difference emerged between the two latter image types (t_(2,20)_ = −0.2, *ns*) (Fig. 3).

For C2 component, post-hoc analyses also showed shorter latencies for P pictures (P: 4411.8 ± 47.7 ms), relative to both NP (4614.8 ± 45.9 ms; t _(2,20)_ = −4.7, *p* < 0.001) and NC pictures (4652.6 ± 45.9 ms; t_(2,20)_ = −4.6, *p* < 0.001), and no difference between the two latter image types (t_(2,20)_ = −1.3, *ns*) (Fig. 3).

### Relationship between behavioral results and SSR

**Figure 4:**
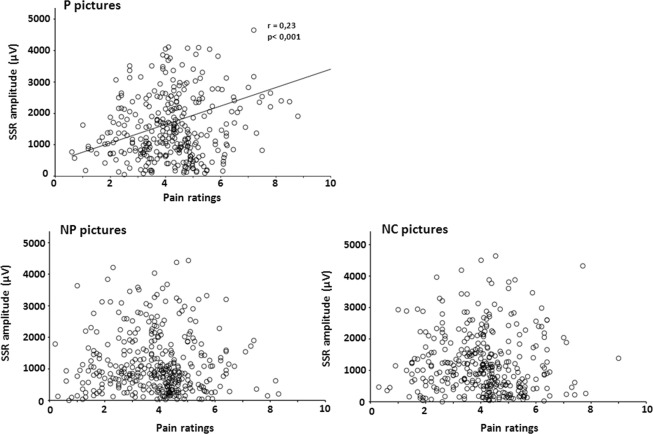
Scatterplots illustrating the relationship between the ratings of the electrical shocks and the C1-C2 amplitudes of the SSR elicited by the three different image types (painful contact (P), non-painful contact (NP) and no contact (NC)). A significant positive correlation was obtained exclusively for the painful pictures.

The potential relationship between pain ratings in the immediate rating conditions (pooled IR1 and IR2) and SSR amplitude was explored with Pearson product-moment correlation. A positive significant, although moderate, correlation between pain ratings and SSR amplitude was observed for painful pictures only (r = 0.23, p < 0.001). No significant correlation emerged for non-painful and non-contact pictures (NP: r = −0.007, ns; NC: r = −0.04, ns) (Fig. 4).

## Discussion

This study confirms that a significant experimental hyperalgesia can be triggered by the observation of images depicting pain. In particular, compared with previous studies using the IAPS database, the pain suggesting images in this study did not depict actual pain states in suffering persons, nor open wounds, bloody or distorted bodies or mutilation scenes, as was the case in previous reports^2,6,19–21^. The present results underscore therefore the robustness of hyperalgesia driven by even ‘mild pain’ observation. They also indicate that the pain-enhancing effect can emerge when the rating of the electric shock immediately followed the shock itself. This suggests that behavioral hyperalgesia results from a rapid perceptual change (less of 1 sec) caused by a rapid modification in the conscious encoding of the nociceptive stimulus, instead of a later bias in memory recollection. This is consistent with electrophysiological results showing that enhancement of pain ratings while seeing pain images are associated with early EEG changes observed at 300–500 ms after the stimulus^2^.

A critical distinctiveness of the present study was the introduction of a second control condition depicting non-painful contacts, which had never been used before in this context. This type of control condition appeared to be crucial, since the mere observation of body parts contacted by objects is able to activate sensory and emotional brain areas^22–24^, this effect thus being a potential confounding factor in previous studies on vicarious pain^16^ or vision-induced hyperalgesia^2,3,20^. This study explicitly disentangles painful from non-painful contact, hence demonstrating that actual or potential pain in the picture definitely appears as the critical factor triggering hyperalgesia, whereas contact *per se* is not.

The magnitude of the hyperalgesic effect, reflected by an increase of subjects’ ratings, could not be directly compared with some of the previous studies^1,3^ due to a lack of access to their non-transformed raw data. The magnitude was in the average (12.9%) range priorly reported in other studies of our laboratory (11%, 15% and 10.5%^2,19,25^) and much higher than that observed in a previous study using a similar set of images^21^. In our study, the hyperalgesic effect produced by pain-depicting picture was robust as well as reproducible across several experimental runs but appeared as ephemeral since this effect was significant only when the rating of the electric shock immediately followed the shock itself. Our results suggest that the robustness of the hyperalgesic effect provoked by pain pictures may not only depend on the short delay separating the image and the subsequent electric shock (<500 msec, see also^7^) but also on the delay between the shock and its sensory rating. The latter effect does not admit a univocal explanation from our data. As shown in Fig. 1, although statistical significance of hyperalgesia was only attained in the ‘immediate response’ trials, the general evolution of pain ratings was similar for rapid and delayed ratings. Since pain ratings were significantly higher and had larger standard deviations in the ‘delayed’ than the ‘immediate’ conditions the lack of effect in the latter might simply reflect statistical underpower linked to worse average/SD ratios.

Pictures depicting painful contacts significantly influenced autonomic reactions, producing earlier and larger SSR responses. The SSR effector limb is a sympathetic noradrenergic pathway originating in the posterior hypothalamus, descending via the intermedio-lateral cell column and, after synapse in paravertebral ganglia, to the sweat glands via unmyelinated cholinergic C-fibers. The SSR is an important component of the orienting response; its amplitude and latency reflect the arousal reaction triggered by a sensory stimulus^8,26^, and have been used to demonstrate pain-related arousal^8,27,28^. This autonomic response is modulated by a set of cortical areas belonging to the limbic/paralimbic systems, including the perigenual cingulate, insular, ventromedial and orbitofrontal cortices^9,15^ and therefore emotional modulation of SSR to our pictures was an expected result^29–31^.

SSR magnitude correlated significantly with pain ratings exclusively in the painful picture condition. The internal variability of SSR values was however very important (see SEM values in the results section) and for a given pain score there was a huge dispersion of possible SSR magnitudes, which lowered the correlation coefficient. Yet, the fact that a significant correlation was attained *exclusively* during the ‘pain pictures’ condition suggests a common driving factor during this condition, able to influence simultaneously SSR magnitudes and pain ratings, which was absent in the two other experimental situations. Since both pain ratings and SSR magnitude are sensitive to the arousal value of a stimulus^32^, one could postulate that increased arousal to ‘pain-relevant pictures’ was responsible for the enhancement of both. Such a simple model finds, however, several difficulties: first, pictures with strictly matched levels of arousal may induce very different levels of pain perception if their contents differ^2^; second, enhanced arousal can also induce a *decrease* in pain ratings, for example when viewing erotic images^20,33,34^. Therefore, although picture-induced arousal may have contributed to increase SSR and pain, it can hardly be considered as the only cause. The intrinsic *content* of the pictures (i.e. the fact that some of them explicitly depicted a painful situation) is likely to have modulated the effect of the ensuing electric shock, since the inherent threat value of a stimulus can modulate both pain sensation and SSR signals^35,36^. Limbic structures influencing emotional perception, such as the amygdala, temporal pole or anterior insula, can respond very rapidly, in less than 300 ms, to complex visual stimuli^37–39^; they have been shown to detect unconscious relationships between stimuli^40^, and can therefore modulate the appraisal of subsequent sensory input. We hypothesize that pain-depicting pictures, via their threat value, induced a change in the subsequent response to shocks arriving 350–500 ms later, endowing them with higher arousal values when coming after painful images, and hence increasing SSR and biasing perception toward more painful levels.

## Conclusion

This study corroborates results from previous experiments describing facilitation of responses to painful stimuli by pain-depicting images. In addition, it shows that such vicarious hyperalgesia is accompanied by enhanced vegetative responses, and demonstrates that seeing actual or potential pain is the critical factor triggering hyperalgesia, while observing contact between innocuous objects and body did not have perceptual or autonomic effects. The signal of bodily threat present in pain-related images appears to enhance the arousal value of a concomitant painful stimulus, modifying both autonomic reactivity and perceptual encoding.

## Methods

### Subjects

Twenty-one healthy volunteers (13 women; mean age 25 years, range 18–42 years) participated to the study. Sample size estimation (G*Power^40^) yielded a minimum of n = 18 participants to obtain an effect size of 0.3, with alpha error conventionally set at 0.05 and power = 0.9. All the subjects were right-handed. No subject was suffering from acute or chronic pain, or was under treatment with painkillers, antidepressants or psychotropic drugs during the experiment. No participant had neurologic or psychiatric disorders. All procedures performed in this study were approved by the national Ethics Committee (Comité de Protection des Personnes Sud-Est III no. 2014-A01280–47) and were in accordance with the 1964 Helsinki declaration and its later amendment. Informed consent was obtained from all individual participants included in the study.

### Stimuli

#### Pictures

**Figure 5:**
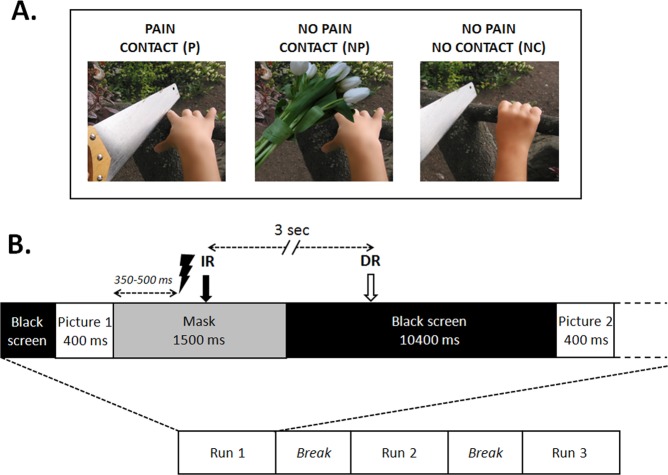
(**A**) Samples of pictures illustrating a painful contact (P), a non-painful contact (NP), and no contact (NC) between an object and a body part. P and NC pictures are reproduced with the permission of Jean Decety, the copyright holder of this image database (Jackson *et al*. 05^16^). NP pictures have been constructed by the authors of the present work by digital manipulation of the “painful” (P) images to replace the aggressive object by a non-aggressive element. (**B**) Schematic representation of the stimulation protocol. IR: Immediate rating, DR: delayed rating.

A series of 96 color pictures were presented via the Presentation® software. Sixty-four of the images were selected from the validated data set used in Jackson *et al*.^16^. These images show a potentially aggressive object (e.g. a knife, a hammer, a saw,) either in direct contact with a human body part (“painful contact” (P), n = 32 images) or at a distance from, and not interacting with, the same body segment (“non-contact” (NC), n = 32 images) (Fig. 5). In addition, a third data set was constructed by digital manipulation of the “painful” (P) images to replace the aggressive object by a non-aggressive element (e.g. a feather, a pencil, a flower) which touched the body segment (“non-painful contact” (NP) n = 32 images). All pictures were perfectly matched in terms of physical characteristics, so that for each “painful contact” image there were corresponding “non-painful contact” and “no contact” pictures of identical size, colour and body segment.

Each experimental run included the three types of images (P, NP, NC), presented in pseudo-random order (examples of images are illustrated in Fig. 5). To avoid persistence of vision, images were masked after 400 ms presentation, by randomly intermixing their own pixels (see below, paragraph 5.4).

#### Electrical stimuli

Electrical stimuli were delivered to the right hand through ring electrodes placed on the thumb with the cathode proximal. The stimuli were generated by an IRES-600 stimulator (Micromed©, Macon, France) and consisted of monophasic constant current pulses of 100 µs duration. Pulse intensity was kept stable during the whole experiment; its intensity was adjusted in each subject previous to the experiment. Using the method of limits, intensity was increased to a level corresponding subjectively to a “pricking and unpleasant clearly painful sensation” matching with a numerical value of 4 on the VAS used in the rest of the study (see paragraph “experimental procedures”). The lowest intensity giving rise to this type of sensation at least 3 times was qualified as “nociceptive threshold”, and used subsequently during the experiment (mean threshold = 17.9 ± 2.6 mA).

### Measure of skin sympathetic response (SSR)

The SSR of each subject was recorded using two Ag-AgCl electrodes with a diameter of 100 mm, localized respectively on the palm and the dorsum of the left hand, previously cleaned with pumicite. The signal was continuously recorded at 512 Hz sampling frequency, amplified × 50,000 and band-pass filtered (non-phase shifting −3 dB cutoff at 0.1–10 Hz; 12 dB rolloff slope) using Brainvision® software and written to hard disk to be processed offline. To allow time-locked averaging digital triggers were sent to the computer simultaneous with each picture presentation, with different labels according to the type of image.

### Experimental procedure

Subjects sat in front of a computer screen. After installation of electrodes for stimulation (on the right hand) and recording (on the left hand), they received instructions and their nociceptive threshold was estimated. Subjects were informed that a series of images would be presented on the screen, coupled to electric shocks; and that they should pay attention to both visual and somatic stimuli and rate the intensity of the latter by drawing a mark on a horizontal 10 cm line. Subjects were not informed that the intensity of stimulation would remain constant during the experiment.

Three consecutive series of images were presented to each subject, each of them containing images of the three types (Painful (P), Non-Painful (NP) and Non-contact (NC)) in random order. Pictures were visible during 400 ms, followed by a 1500 ms mask constructed by randomly intermixing the image’s own pixels, and then by a black screen lasting 10,400 ms. An electric shock was delivered at the end of each picture presentation between 350 and 500 ms after the beginning of the mask.

Subjects evaluated the intensity of the electric stimulation by drawing a mark on a horizontal line corresponding to a visual analogue scale (VAS). The line (10 cm long) was marked “no sensation” on its left end, “maximal pain” on its right end. Some points were placed on the scale to help the ratings, at approximate numerical values of 0 (no sensation at all), 1 (you feel a sensation, but it is not painful), 2 (you feel a strong sensation, but it is not painful), 3 (starts to be painful, but is a very mild pain), 8 (very painful and demanding some effort to tolerate), 10 (the worst imaginable pain). The indications drawn by the participants after each shock allowed therefore to estimate their subjective perception level relative to the marks pre-defined on the line. In 2 of the 3 runs, the subject was asked to rate the sensation immediately after the electrical stimulus (IR: ‘immediate rating’ conditions, two blocks: IR_1 and IR_2), while in the other the intensity estimates were provided 3 seconds later (DR: ‘delayed rating’ condition). Sixteen images of each category (P, NP and NC) were presented in one IR block (a total of 48 images in the block). Images were different in the two IR blocks. For the DR block, 48 images were randomly chosen among the previous ones. In the DR condition, a sign appeared on a computer screen, indicating to the subject when he could perform the intensity rating. The order of presentation of the DR run among the two other runs was chosen randomly. Several training trials were performed to let subjects learn how to use the rating scale. To minimize disruption of the SSR due to other external stimuli, each subject wore earplugs during the entire experiment. Figure 5 outlines graphically the experimental procedure.

### Data analysis

#### Behavioral data

Ratings collected during the experiment (measured in centimeters from the leftmost end of the scale) were grouped according to the type of the picture presented, and analyzed statistically using JASP (V 0.9) software. Data on perceptive ratings underwent 2-way, repeated-measures analysis of variance (ANOVA) with “image type” (P *vs*. NP *vs*. NC) and “block type” (immediate 1 (IR_1) *vs* immediate 2 (IR_2) *vs* delayed (DR)) as factors. When appropriate, the Geisser-Greenhouse (G-G) procedure was applied to correct degrees of freedom. A level of p < 0.05 after correction was accepted as statistically significant. Post-hoc comparisons (t-tests Bonferroni corrected) were performed when ANOVA yielded significant main results.

#### Sympathetic skin response (SSR)

Analysis of SSR was made through BrainVision® software. The continuous electrodermal signal was cut into epochs of 9000 ms (from 1000 ms before the appearance of the image to 8000 ms post-stimulus) and averaged according to picture type (P, NP and NC) for each subject. For illustration purposes, grand-averages of SSR of all subjects were computed for each picture type; however, statistical analysis were performed on individual (ie not grand-averaged) data. Amplitudes and latencies of the SSR peaks were measured trial by trial in the three blocks. The two main SSR components were labeled respectively “C1” (the first negative deflection peaking at about 3000 ms) and “C2” (positive deflection peaking at about 4500 ms) (Fig. 2). Amplitudes were measured from peak-to-peak, between C1 and C2 maxima. Habituation on SSR across blocks was assessed by one-way repeated-measures ANOVA on amplitudes with the “block order” as factor (block 1 vs block 2 vs block 3). As no order effects were observed for all the image categories (P: *F*_(2,20)_ = 0.8; ns; NP: *F*_(2,20)_ = 0.9; ns; NC: *F*_(2,20)_ = 0.6; ns), all the blocks were pooled together to perform the following analyses.

Amplitude and latency SSR values were each submitted to a one-way repeated-measures ANOVA, with ‘image type’ (P, NP and NC) as within subject factor. When appropriate, the Geisser-Greenhouse (G-G) procedure was applied to correct degrees of freedom. A level of p < 0.05 after correction was accepted as statistically significant. Post-hoc comparisons (t-tests, Bonferroni corrected) were performed when ANOVA yielded significant main results.

Pearson Product-moment correlations between subjective ratings and SSR amplitudes were calculated using JASP (Version 0.9) software. In text and tables, values are given as means ± SEM.

## Data Availability

The datasets generated during and/or analysed during the current study are available from the corresponding author on reasonable request.
